# Investigating the effective factors in creatinine changes among hemodialysis patients using the linear random effects model 

**Published:** 2015

**Authors:** B Shabankhani, A Kazemnezhad, F Zaeri

**Affiliations:** * PhD Candidate in Biostatistics, Faculty of Medical Sciences, Tarbiat Moodares University, Tehran, Iran; ** Department of Biostatistics, Faculty of Medical Sciences, Tarbiat Modares University, Tehran, Iran; *** Department of Biostatistics, Shahid Beheshti University of Medical Sciences, Tehran, Iran

**Keywords:** creatinine changes, hemodialysis patients, linear random effects model

## Abstract

**Background and objectives:**Out of 10 apparently healthy humans, one was somewhat suffering from one of the types of renal disease. Hemodialysis is known as the most applicable method of taking care of this group of patients. In addition, serum creatinine is an important mark in the performance of kidneys. The aim of the present study was to investigate the effective factors in creatinine and its effect on the performance of kidneys.

**Materials and methods:** The present study is a longitudinal experiment in which 500 participants were randomly selected from the hemodialysis patients in Mazandaran Province. Creatinine variable was considered as the longitudinal responding variable, which was measured 3 times per year over a period of 6 years. The random effects model was also considered the most appropriate model for the collected data.

**Results:**The total mean value of creatinine was 1.62 ± 0.49, among men 1.69 ± 0.46 and among women 35.1 ± 0.49. Variables of weight (p<0.001), age of disease diagnosis (p<0.001), time (p<0.001), gender (p<0.005), and cardiovascular diseases were significant and had effects on the trend of creatinine changes among the hemodialysis patients. Creatinine mean value had an increasing trend.

**Conclusion:**Blood creatinine had a significant effect on the performance of kidneys, and the identification of variables that affected the creatinine level was highly helpful in controlling the performance of the kidneys. The results of most studies conducted on hemodialysis patients indicated that by measuring and controlling variables like weight, tobacco consumption, and control of related diseases like blood pressure could predict and control creatinine changes precisely.

## Introduction

Global statistics indicate that there are 550 million renal patients. That is, out of 10 apparently healthy people, one suffers from a type of renal diseases. Nowadays, hemodialysis is known as the most applicable method to provide healthcare to this group of patients. About 70% of the renal patients are treated through this method [**1**]. In the USA, about 10 to 15% of the adults suffer from renal failure. In 1988, approximately 3,200,000 patients with renal disease were going through their final stages, 72% of whom were treated through hemodialysis [**2**]. Iran’s Ministry of Health has also reported the prevalence of this disease, 2.5 out of 1000 people, and predicted its annual growth to be 12% in 2006. The kidney transplant is the best treatment for this disease. However, considering the low statistics of kidney transplant in Iran, i.e. 24 cases out of 1 million, hemodialysis is still the most common method to prevent the disease. The hemodialysis of the patients imposes high expenses on the health system such that in the current year, the Health Deputy of the Ministry of Health has spent 20 billion tomans (one US dollar equals 3,200 tomans – free market rate of exchange) on the filters of dialysis devices, and the annual expense for the hemodialysis of a patient has been estimated to be 18 million tomans [**3**,**4**]. Due to the high prevalence of this disease, a large body of research has been conducted on this issue. However, most of them have utilized classical statistical methods, which were not flawless due to the type of relationship between the thematic variables. The present study was an attempt to analyze the data by using advanced statistical analysis methods. An important marker of performance of kidneys is serum creatinine, and in most studies, it has been introduced as the marker of kidneys’ performance [**5**-**8**].

In medical studies, responses need to be measured frequently in order to evaluate more precisely the intervention methods. However, in some cases, the researchers have to deal with correlated responses and in most cases, this correlation is sensitive to time, i.e. the intensity of the correlation increases or decreases with time. Due to this correlation, typical methods like the method of the least square means that it cannot be employed. That is why methods that are dealing with correlated responses have nowadays gained a high significance. For instance, one can refer to changes in markers related to diseases over time such as frequent evaluation of CD4 in HIV+ patients to predict AIDS [**9**-**11**]. Nowadays, studies in which the effects of time are considered are classified into types such as trend study, cohort study, etc. In a trend study, samples of different groups of the statistical population were investigated at different times. This type of studies provide the variables of only one group or society. Trend studies can be useful in describing long-term changes in a society. Cohort studies include a group of individuals who are correlated in a way or a group of individuals who have experienced an important life experience in a specific period, like people who have married in the same year, those who were born in the same hospital, etc. Therefore, any study in which there are values of a feature in two or some periods of time is considered a cohort study (e.g., the rate of non-academic study among freshmen and seniors). This type of research is an attempt to find a known cohort effect. Some studies contain data that are measured several times over a certain period of time, this type of data being called longitudinal data. The present research is a historical cohort study in which the response variable is a longitudinal one. In longitudinal studies, the same group of respondents is examined at different times. In other words, the response variable is measured several times over a certain period of time in such studies [**12**-**14**]. Two important advantages in analyzing longitudinal data include measuring the effect among the individuals and measuring the changes of each individual in relation to time. Since every participant enters the study with his/ her own specific characteristics, the random effects model was employed. Due to specific characteristics of every individual, regression coefficients for each person was different from others.

The present study is an attempt not only to conduct a collective analysis but also to measure the individual effects by using longitudinal methods and the individuals’ characteristics and frequently measuring serum creatinine. The present study was mainly aimed at examining the change in the creatinine variable and the effect of the effective variables in changes of this variable.

## Materials and methods

The present study was a historical cohort research in which the data related to 500 hemodialysis patients were utilized. They were randomly selected from among hemodialysis patients of Mazandaran Province. The required data were collected over a period of 6 years.

The blood creatinine variable was considered the response variable in the present study, and its values were extracted 18 times during 2007 and 2013 from the individuals’ profiles.

Other variables included gender, age, marital status, education, smoking background, cause of renal disease, family support, cardiovascular diseases, weight, age of disease diagnosis, age of dialysis beginning.

Since data were longitudinal, the independence condition of it was not met. Therefore, a simple statistical analysis was first conducted in order to describe the variables. Then the longitudinal condition of the data was examined, and after it was ascertained, in order to evaluate the model it was tested by using all helping variables. Afterwards, the final embedding of the model was carried out by using significant variables of the previous phase. The response variable in this section was creatinine, and random effects model was chosen as the appropriate model for the collected data. In this model, the response variable is a function of helping variables with regression coefficients that are different from one subject to another. This difference is due to non-measured factors like genetics and natural factors. By using this method, intrapersonal changes of the response variable can be measured over time. Data analysis was conducted by using SPSS 20.0 and STATA 13.0.

## Results

In the present study, 53.6% of the patients were men and 46.4% were women. The mean age of the hemodialysis patients was 60 ± 16.84 years. While the mean age of male patients was 60 ± 17.4, it was 59 ± 16.18 for women. Moreover, the mean age of dialysis beginning was calculated to be 56 ± 17.43; for men it was 56 ± 18.07 and for women 55 ± 16.70. The minimum and maximum ages of dialysis beginning were 3 and 90 years, respectively. While the maximum age of dialysis beginning was the same for both groups, the minimum age was reported to be 15 and 3 years for men and women, respectively. Moreover, the results indicated that the highest rate of mortality (54%) was related to patients whose cause of dialysis was unknown.

In the present study, the longitudinal variable of creatinine was measured 3 times a year, having a total of 18 times. The total mean value of creatinine was 1.62 ± 0.49; for men and women it was 1.69 ± 0.46 and 1.53 ± 0.49, respectively. The highest level of calculated creatinine during the study was related to patients whose dialysis cause was unknown (1.79 ± 0.41) and the lowest level was related to those whose dialysis cause was diagnosed to be the stone (1.55 ± 0.52). The mean creatinine among patients who had family support was significantly less than those without it (p<0.01).

**Diagram 1:**
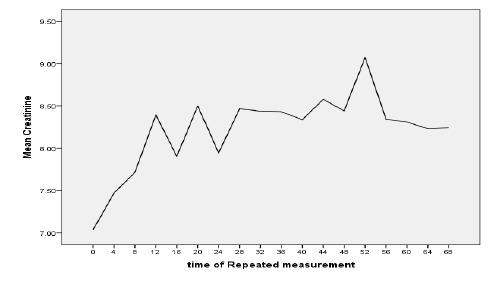
Measured mean creatinine during the 18 times of measurement

**Table 1** presents the data related to creatinine amounts measured during the study. **Diagram 1** also shows the data related to creatinine amounts measured during the study. The biggest difference was observed in the beginning of the study. In general, the collected data indicate that creatinine changes have a positive growth in relation to time.

**Table 1:** The measured creatinine amounts after 18 times of measurements during the study

Time of Measurement	Mean	SD	Time of Measurement	Mean	SD
1	7.03	2.85	10	8.43	2.49
2	7.47	3.15	11	8.33	2.24
3	7.71	2.96	12	8.57	2.47
4	8.39	2.87	13	8.44	2.39
5	7.90	2.96	14	9.07	2.70
6	8.50	3.04	15	8.34	2.61
7	7.94	2.43	16	8.30	2.51
8	8.46	2.35	17	8.23	2.70
9	8.43	2.60	18	8.24	2.99

The following diagram indicates the course of the disease, which was drawn by using a random sample of 20 individuals who were randomly selected from among the patients. As seen, a different interception of the samples totally justifies the individual effects. Moreover, different trends that are observed for each sample in continuation indicate the significant effect of time in the present study.

**Diagram 2:**
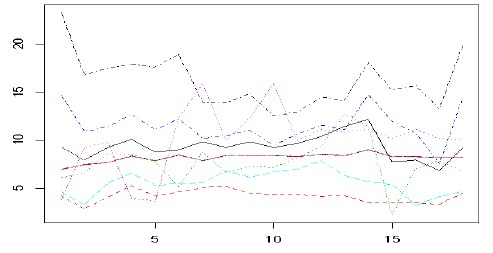
Creatinine course in a 20-individual sample of patients treated with hemodialysis

The embedding of the model was carried out in the presence of all variables of the study. Variables of weight, age of disease diagnosis, time of repetition, blood pressure, gender, and cardiovascular diseases were significant. The results indicated that the mean creatinine increased with 0.049 per one kilogram of weight gain (p<0.001). Moreover, with one year increase in age of diagnosis, the mean creatinine level decreased with 0.031 (p<0.001). With the passing of every 4 months, the mean blood creatinine level increased with 0.013 (p<0.001). The mean creatinine level for women was 0.53 less than that of men (p<0.005). Moreover, the mean creatinine level among patients whose disease cause was high blood pressure was 0.670 times higher than that of those whose disease cause was reported to be diabetes.

The mean creatinine level among patients who did not have a heart disease history was 0.305% higher than that of those who had such a history. The results indicated that 65.6% of the observed changes were due to random effects. This point justifies the use of the random effects model for the collected data.

**Table 2:** The results of the final analysis of the embedding of the model

Variable	Category	Coefficients	Std.	Sig.	Confidence Interval of 95%	
Weight	-	0.049	0.008	0.000	0.066	0.031
Age of Disease Diagnosis	-	-0.031	0.006	0.000	-0.019	-0.043
Time Effect	-	0.013	0.000	0.000	0.015	0.012
Job	Unemployed	-0.374	0.445	0.400	0.498	-1.247
	Office worker	-0.674	0.585	0.249	0.472	-1.821
	Farmer	-0.446	0.585	0.446	0.700	-1.593
	Retired	-0.689	0.546	0.207	0.382	-1.761
	Other	0.451	0.551	0.413	1.532	-0.629
	Housekeeper (reference)					
Education	Low Literate	-0.136	0.237	0.565	0.329	-0.602
	Diploma	0.110	0.344	0.749	0.784	-0.564
	MA and Upper	0.230	0.482	0.633	1.176	-0.715
	Illiterate (reference)					
Disease Cause	Blood Pressure	0.670	0.418	0.109	1.491	-0.150
	Stone and Blockage	0.129	0.620	0.835	1.344	-1.085
	Renal Cyst	-0.102	0.446	0.819	0.773	-0.977
	Birth Defects	0.285	0.256	0.265	0.788	-0.216
	Other Causes	0.387	0.475	0.416	1.318	-0.544
	Diabetes (reference)					
Gender	Female	-0.781	0.451	0.083	0.103	-1.665
	Male (reference)					
Smoking	Yes	-0.112	0.277	0.685	0.430	-0.655
	No (reference)					
Cardiovascular Disease	Yes	0.305	0.197	0.123	0.692	-0.082
	No					
Variance of Random Effect	-	6.409	0.917	0.000	8.208	4.610

## Discussion

The results of the present study indicated that variables of gender, age of disease diagnosis, cardiovascular disease, and blood pressure have a significant effect on blood creatinine changes. A review of the studies conducted on this issue and their comparison indicated that there are more significant variables. However, it is clear that longitudinal studies depend on time. Therefore, values are measured over time, and that is why data recorded in the forms are different from the collected ones because they have not been recorded at the same time.

Luigi et al. conducted a study in order to predict serum creatinine among 179 hemodialysis patients. The results of their study indicated that there was a significant difference between CRP and increased changes in serum creatinine. They employed multivariate linear regression and categorized the patients according to their gender and diabetes stage. They concluded that age could be considered a predictive factor for serum creatinine in men. Another objective of their study was to determine the potential relationship between serum creatinine, senility, gender, and dialysis efficiency. Since creatinine is the product of the muscles and the body’s mechanism that declines in older patients, and probably due to less development of muscle mass in women, age, with its approved effect on gender, has a higher importance in predicting serum creatinine. The results of their study are in agreement with those of the present study [**5**].

A study was conducted on 7,719 adult hemodialysis patients in the USA, in order to investigate their mortality risk and changes in their diet criteria. The values measured of serum albumin and serum creatinine before the experiment were remarkably related to mortality risk which in turn had a reversible relationship with serum creatinine in the beginning of the study. There was a strong reversible relationship between the number of neutrophils and serum albumins and between the number of neutrophils and creatinine [**15**]. In another study conducted by Kaysen et al. in order to compare the effects of creatinine and albumin among 364 patients with hemodialysis, the results indicated that creatinine changes over time were not significant. Moreover, age, gender, race, and diabetes were reported to be significant, which is in line with the present study [**6**]. Ramer et al. conducted a retrospective study on 2,131,248 adults in Pennsylvania. Among these adults, 6,657 had severe hemodialysis. The results of their study indicated that 43% of those with severe hemodialysis died one year after admission. In comparison with the present study in which death probability was 17% in the first year, a significant difference was observed, which could be related to the fact that the participants in Ramer’s study were going through the final stages of their disease. Moreover, variables of age, gender, race, and insurance status in Ramer’s study and other studies conducted in the USA were reported as significant variables with survival of this group of the patients, these results being in line with those of the present study [**16**].

Another study was conducted in order to predict premature death among diabetic patients suffering from hemodialysis, in which risk factors related to mortality within 3, 6, and 12 months were investigated. The relationship between age and heart diseases was reported to be significant [**17**]. Speckman reported that the main risk factors in patients with hemodialysis are diabetes and cardiovascular diseases. That is why diabetes was considered the basic cause of disease in the present study [**18**].

Johansen et al. [**19**] studied 54 hemodialysis patients. In their study, they measured the performance of the patients’ kidneys 4 times in a year. Changes in repetition sizes were investigated with regulations for differences of age, gender, race, and diabetes status basis. No remarkable change was observed in body weight, fat mass, and lean body mass. The reason for the different results of this study and the present study can be attributed to the short period of follow-up in Johansen’s study compared to the present one [**19**].

Oomichi et al. conducted a study in order to investigate the effect of regular control of blood sugar in the survival of diabetic patients with chronic hemodialysis. In the beginning of hemodialysis, there was no significant difference in the admission age, dialysis duration, blood pressure, proportion of heart patients, and serum creatinine level in the three groups. The most important factor in the survival of this group of the patients was reported to be the quality of healthcare provided to these individuals [**20**].

Another study was conducted in order to investigate the quality of life among 90 patients with dialysis in Malaysia. The mean age of the patients was 7.94 ± 14.1 and the maximum occupational frequency was related to the unemployed with 71.1%. The main cause of disease was unknown. The mean weight was 57.7 and the mean duration of dialysis was 55 ± 39 months. Diabetes, high blood creatinine, and decreased calcium were introduced as the significant variables of the study. The mean serum creatinine was 3.7 [**21**].

Another study was conducted in order to investigate the results of dialysis simultaneously in 7 countries including France, Germany, Italy, Japan, Spain, the UK, and the USA. In this study, 24,392 patients were selected from 327 centers in these countries. The study was conducted by using a longitudinal method. In this study, the patient’s age, gender, and diabetes were introduced as the cause of end stage renal disease (ESRD) [**22**].

While hemodialysis to some extent enhances life expectancy among renal patients who are going through final stages, the quality of life among such patients has been reported to be low. That is why related effective variables are highly significant.
